# Anti-HER2 induced myeloid cell alterations correspond with increasing vascular maturation in a murine model of HER2+ breast cancer

**DOI:** 10.1186/s12885-020-06868-4

**Published:** 2020-04-28

**Authors:** Meghan J. Bloom, Angela M. Jarrett, Todd A. Triplett, Anum K. Syed, Tessa Davis, Thomas E. Yankeelov, Anna G. Sorace

**Affiliations:** 1 Department of Biomedical Engineering, The University of Texas, Austin, TX USA; 2 LiveSTRONG Cancer Institutes, The University of Texas, Austin, TX USA; 3 Department of Oncology, The University of Texas Dell Medical School, Austin, TX USA; 4 Diagnostic Medicine, The University of Texas, Austin, TX USA; 5 Oden Institute for Computational and Engineering Sciences, The University of Texas, Austin, TX USA; 6 Department of Radiology, The University of Alabama, Birmingham, AL USA; 7 Department of Biomedical Engineering, The University of Alabama, Birmingham, AL USA; 8 O’Neal Comprehensive Cancer Center, The University of Alabama, Birmingham, AL USA

**Keywords:** Angiogenesis, Tumor associated macrophages, Herceptin, Trastuzumab, BT474

## Abstract

**Background:**

Therapy targeted to the human epidermal growth factor receptor type 2 (HER2) is used in combination with cytotoxic therapy in treatment of HER2+ breast cancer. Trastuzumab, a monoclonal antibody that targets HER2, has been shown pre-clinically to induce vascular changes that can increase delivery of chemotherapy. To quantify the role of immune modulation in treatment-induced vascular changes, this study identifies temporal changes in myeloid cell infiltration with corresponding vascular alterations in a preclinical model of HER2+ breast cancer following trastuzumab treatment.

**Methods:**

HER2+ tumor-bearing mice (*N* = 46) were treated with trastuzumab or saline. After extraction, half of each tumor was analyzed by immunophenotyping using flow cytometry. The other half was quantified by immunohistochemistry to characterize macrophage infiltration (F4/80), vascularity (CD31 and α-SMA), proliferation (Ki67) and cellularity (H&E). Additional mice (*N* = 10) were used to quantify differences in tumor cytokines between control and treated groups.

**Results:**

Immunophenotyping showed an increase in macrophage infiltration 24 h after trastuzumab treatment (*P* ≤ 0.05). With continued trastuzumab treatment, the M1 macrophage population increased (*P* = 0.02). Increases in vessel maturation index (i.e., the ratio of α-SMA to CD31) positively correlated with increases in tumor infiltrating M1 macrophages (R = 0.33, *P* = 0.04). Decreases in VEGF-A and increases in inflammatory cytokines (TNF-α, IL-1β, CCL21, CCL7, and CXCL10) were observed with continued trastuzumab treatment (*P* ≤ 0.05).

**Conclusions:**

Preliminary results from this study in a murine model of HER2+ breast cancer show correlations between immune modulation and vascular changes, and reveals the potential for anti-HER2 therapy to reprogram immunosuppressive components of the tumor microenvironment. The quantification of immune modulation in HER2+ breast cancer, as well as the mechanistic insight of vascular alterations after anti-HER2 treatment, represent novel contributions and warrant further assessment for potential clinical translation.

## Background

Approximately one in five cases of breast cancer overexpress the human epidermal growth factor receptor 2 (HER2) [1]. Patients with HER2+ breast cancer have shorter disease free survival, decreased overall survival rates, and greater metastatic potential than HER2- patients [2, 3]. The current standard-of-care therapy for HER2+ breast cancer is cytotoxic chemotherapy in combination with trastuzumab, a targeted monoclonal antibody that binds to the HER2/neu receptor. Treatment with trastuzumab induces cell cycle arrest and inhibits cancer cell proliferation [4, 5]. Trastuzumab can also downregulate the expression of HER2 by promoting receptor internalization and degradation [6]. As a secondary effect, trastuzumab has been shown to alter the characteristics of tumor-associated vessels through increasing vascular maturation and stabilization in HER2+ tumors [7–10]. Such changes can subsequently enhance the efficacy of combination therapies and improve treatment response in multiple types of cancer including breast, colon and lung [7, 11, 12]. With less than half of patients responsive to neoadjuvant therapy in HER2+ breast cancer, further understanding of vascular changes that have potential to enhance therapeutic response may provide a clinically translatable benefit [13].

Trastuzumab-induced vascular maturation is partly attributed to the decrease in vascular endothelial growth factor (VEGF) secretion, but the exact mechanisms have not fully been elucidated [7–9]. Clinical data evaluating progression-free survival from anti-angiogenic therapies targeting VEGF signaling pathways (such as bevacizumab) in breast cancer conflict between the United States and the European Union, but neither finds a significant improvement in overall survival rates [14–17]. One mechanism by which tumor cells can become resistant to anti-VEGF therapy is through the recruitment of pro-angiogenic, immunosuppressive myeloid cells [18, 19]. Myeloid cells are non-lymphoid immune cells that influence the development of tumor vasculature through the secretion of pro- and anti-angiogenic factors [20, 21]. When anti-VEGF therapies are administered at high doses or for a prolonged period of time, tumor vasculature is overly pruned and leads to tumor hypoxia and upregulation of hypoxia inducible factors (HIFs) [19, 22]. HIFs drive recruitment of tumor associated macrophages (TAMs), myeloid derived suppressor cells (MDSCs), and prevent maturation of dendritic cells [20, 23, 24]. Angiogenesis is then driven by myeloid cells through both VEGF dependent and independent pathways enhancing tumor progression [18, 19]. However, when tumor vasculature is normalized, tumor hypoxia decreases, and the balance of angiogenic factors in the tumor microenvironment can be restored through the reprogramming of the myeloid cell population—further stabilizing the vasculature and promoting antitumor responses [25, 26]. Both clinical and preclinical studies show that treatment with trastuzumab alters immune infiltration (including myeloid populations) in HER2+ tumors [27–29]; however, to our knowledge, a detailed immune panel in HER2+ breast cancer identifying changes in myeloid populations to trastuzumab has not been conducted.

Our previously acquired immunofluorescence data revealed an increase in macrophage population after trastuzumab treatment in the BT474 human-derived cell line animal model of HER2+ breast cancer [29]. TAMs are a significant component of the breast tumor microenvironment and of particular interest due to their influence in tumor progression and response to treatment [30, 31]. TAMs largely polarize towards two phenotypes, “classical” (M1) or “alternative” (M2) depending on the stimuli present in the tumor microenvironment and have been shown to either enhance (M1) or suppress (M2) anti-tumor immune responses [32, 33]. Macrophage differentiation towards the M2 phenotype are driven by interleukin 4 (IL-4) and interleukin 13 (IL-13); and promote angiogenesis by secreting VEGF, basic fibroblast growth factor, and several matrix metalloproteases (MMPs) [34–36]. Macrophages are polarized towards an M1 phenotype by lipopolysaccharide and interferon gamma (IFN-γ). They have a lower angiogenic potential than M2 macrophages and promote anti-tumor immunity through secretion of pro-inflammatory cytokines and increased antigen presentation ability [34–37].

In this study, we sought to gain insight into the mechanisms of improved tumor vascularization and heightened windows of anti-tumor immunity after administration of trastuzumab. First, we evaluated immune modulation by quantifying myeloid cell populations, including M1 and M2 macrophages, following trastuzumab treatment in a xenograft model by flow cytometry. Secondly, we validated concurrent vascular alterations using quantitative histology. Finally, we identified changes in tumor cytokines and chemokines relating to immunity and angiogenesis through a multiplex cytokine detection assay.

## Methods

### Cell culture

BT474 breast cancer cells were purchased from ATCC (Manassas, Virginia). Cells were cultured in improved minimal essential medium (IMEM, Invitrogen, Carlsbad, CA) supplemented with 10% FBS, 1% penicillin/streptomycin, and 20 μg/mL insulin. Cells were grown at 37 °C with 5% CO_2_. Cells were cultured to 70–80% confluency and all cell counts were determined by the Countess II FL automated cell counter (Thermo Fisher Scientific Inc., Waltham, MA).

### Animal procedures

**Fig. 1:**
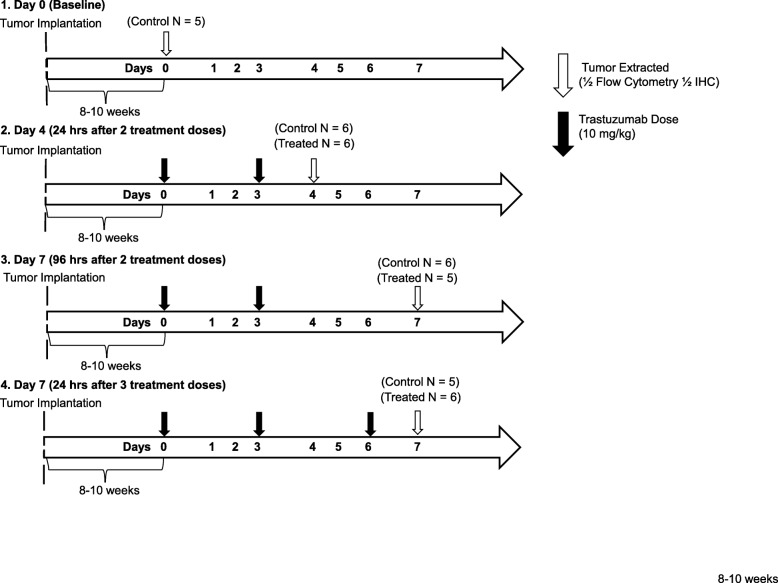
Outline of experimental procedure and treatment schedules. **a** BT474 HER2+ tumor-bearing mice were divided into four treatment groups. Tumors were extracted from group 1 on Day 0 without treatment. Group 2 and 3 received two doses of saline (Control) or trastuzumab (Treated) on Days 0 and 3 and tumors extracted on Day 4 (group 2) and Day 7 (group 3). Group 4 received three doses of saline or trastuzumab on Days 0, 3 and 6 and had tumors extracted on Day 7

All procedures were approved by The University of Texas at Austin’s institution animal care and use committee (IACUC). Female nude athymic mice (*N* = 56) were purchased from The Jackson Laboratory (Bar Harbor, ME) at 3–4 weeks of age and were maintained with microisolator cages with ventilated racks in a standard light cycle facility with enrichment. After a one-week acclimation period, mice were subcutaneously implanted with a 0.72 mg, 60-day release, 17β-estradiol pellet (Innovative Research of America, Sarasota, FL) in the nape of the neck. 24 h later, 10^7^ BT474 breast cancer cells in 100 μl serum-free IMEM media with 30% growth factor-reduced Matrigel were injected subcutaneously into the right flank of the mouse. Tumors were grown to approximately 250 mm^3^ (estimated 8–10 weeks) at which point the animals were entered into the study. Animals were randomly sorted into treatment groups and administered either trastuzumab (10 mg/kg) or saline on three different days (Days 0, 3 and 6) via intraperitoneal (IP) injection during a week-long treatment plan. Mice were euthanized for tissue processing on days 0 (*N* = 5), 4 (*N* = 6 control, N = 6 treated) and 7. Mice taken down on Day 7 either received two total doses of treatment on Days 0 and 3 (N = 6 control, N = 5 treated) or three total doses of treatment on Days 0, 3 and 6 (N = 5 control, N = 6 treated) (Fig. 1). All euthanasia is performed by dual methods with sustained isoflurane followed by cervical dislocation.

### Spleen disaggregation

The spleen was excised and immediately placed on ice in RPMI 1640 serum free media (Caisson Laboratories, Smithfield, UT). All further incubation periods and reagents were at 4 **°**C. The spleen was then homogenized to a single cell suspension and diluted in flow wash buffer (FWB, PBS + 1% FBS + 5 mM EDTA). Cells were resuspended in 1× red blood cell (RBC) lysis buffer (Biolegend, San Diego, CA) for 3 min. The cell suspension was washed in FWB and filtered through a 70 μM strainer.

### Tumor disaggregation

After tumor excision, the tissue was cut at the longest cross-section and half was placed in a tissue cassette and incubated in 10% neutral buffered formalin (Fisher Scientific International Inc., Pittsburgh, PA) for 48 h. Samples were then transferred to 70% ethanol and prepped for immunohistochemistry (IHC) staining. The other half of the tumor was cut into 2–3 mm pieces and placed in 0.15% collagenase type IV (Worthington Biochemical Corporation, Lakewood, NJ) with 5 mM Ca^2^ at 1 mL/100 mg of tissue. Mechanical dissociation was performed using the gentleMACs dissociator (Miltenyi Biotec, Bergisch Gladbach, Germany). Samples were incubated while rotating at 37 °C for 45 min. After digestion, the suspension was washed in FWB and resuspended in 3 mL of 1× RBC lysis buffer for 3 min on ice. Cells were washed in FWB and filtered through a 70 μM strainer.

### Flow Cytometry

Cells harvested from the spleen were used for single color and multi-color staining controls. Control and tumor samples were resuspended at a concentration of 10^6^ cells/100 uL in FWB in round bottom polystyrene tubes. Cells were stained with the following antibody fluorophore conjugates: CD45-PerCP/Cy5.5, CD11c-Pacific Blue, CD11b-AF700, MHCII-APC/Cy7, Ly6c-BV510, Ly6g-APC, CD38-FITC (Biolegend, San Diego, CA), F480-PE/Cy7 (Tonbo Bioscences, San Diego, CA), CD206-PE (R&D Systems, Minneapolis, MN) and propidium iodide (PI, Enzo Life Sciences, Farmingdale, NY). All antibody staining took place for 30 min at 4 **°**C in the dark. For all experiments, cells were acquired on the BD LSRII Fortessa flow cytometer. Compensation and sequential gating was performed with FlowJo software (FlowJo LLC, Ashland, OR). Populations of myeloid cells that were identified included macrophages, dendritic cells, granulocytic myeloid derived suppressor cells (G-MDSC), monocytic myeloid derived suppressor cells (M-MDSC) and M0, M1 and M2 macrophage phenotypes (Supplementary Fig. S1).

### Immunohistochemistry

Formalin fixed tumor sections were embedded in paraffin. Tumors were then sliced into 4 μM sections and stained for the following: hematoxylin and eosin (H&E), mouse anti-CD31, mouse anti-α-smooth muscle actin (α-SMA, Abcam, Cambridge, UK), mouse anti-F480 (Invitrogen, Carlsbad, CA), and human anti-Ki67 (R&D Systems, Minneapolis, MN). Immuno-stained slides were scanned (20×, 0.495 μm/pixel) with the Aperio ScanScope (Leica Microsystems, Wetzlar, Germany). Automated segmentation of tissue structure was completed using custom MATLAB algorithms (MathWorks Inc., Natick, MA). All images were uniformly segmented based on thresholds determined from positive and negative controls for each stain except for regions of necrosis, which were determined manually from H&E staining. Images were registered to the corresponding H&E stained image from that sample. For registration, images were converted to grayscale. Transformations applied to images consisted of translation, rotation and scale (similarity) based on intensity differences. A viable tissue mask was defined as total tumor area minus necrotic area. Macrophage infiltration (F4/80) was defined as the percent of positive stain per viable tissue area. Microvessel density (CD31) and vascular smooth muscle coverage (α-SMA) were calculated as the number of vessels per mm^2^ of tumor tissue. The vessel maturation index was evaluated as the ratio of α-SMA coverage to microvessel density [38]. To confirm treatment response, proliferation (Ki67) was defined as the percent of total tumor nuclei positively stained. All codes are available upon request.

### Cytokine detection assay

Tumor bearing mice (*n* = 10) were treated with either three total doses of trastuzumab (10 mg/kg) or saline for 1 week on Days 0, 3 and 6. On Day 7, tumors were excised and flash frozen with liquid nitrogen in Optimal Cutting Temperature compound (Tissue-Tek; Sakura Finetek USA). Tumors were lysed in Cell Lysis Buffer 2 (R&D Systems, Minneapolis, MN). Samples were concentrated using Pierce Protein Concentrator PES 3 K (Thermo Fisher Scientific Inc., Waltham, MA), and original sample protein concentration was determined using the NanoDrop 2000 (Thermo Fisher Scientific Inc., Waltham, MA). The multiplex assay was conducted using the Bioplex 200 (BioRad Laboratories, Hercules, CA). Samples were incubated with premixed beads targeted to 12 different analytes according to the Mouse Magnetic Luminex Assay (R&D Systems, Minneapolis, MN) specifications. Protein concentrations were determined using corresponding standard curves for each analyte (Supplementary Fig. S2).

### Statistical analysis

Statistical analysis was conducted using MATLAB (MathWorks Inc., Natick, MA). Statistical differences between treatment groups of flow cytometry, immunohistochemistry, and cytokine data were determined using a nonparametric Wilcoxon rank sum test. All data is presented as mean ± standard error with *P* ≤ 0.05 indicating significance. Linear regression parameters and Pearson correlation coefficients were determined for flow cytometry and histology comparisons. Tumors were eliminated from analysis if viability was < 50% due to prolonged incubation as determined with live/dead stain in flow cytometry (*N* = 4), or if ≥4 myeloid cell populations were significant outliers (*N* = 3). Outliers were defined as a value more than three absolute deviations from the median.

## Results

### Animals

All animals had no significant changes in weight loss or adverse events

### Macrophage infiltration increases transiently after trastuzumab treatment

**Fig. 2:**
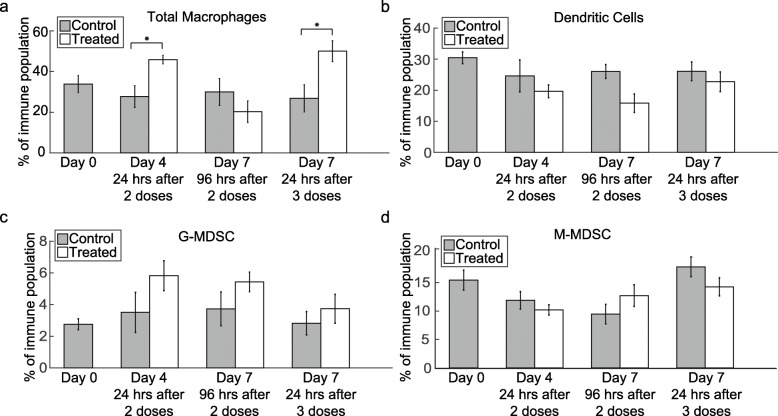
Macrophage infiltration increases 24 h after administration of trastuzumab. **a** Percent of total macrophages in the tumor immune population is shown, revealing a significant increase in macrophage infiltration on Day 4 (*P* = 0.02) and Day 7, 24 h after treatment (*P* = 0.03) compared to control tumors. Percent of **b** dendritic cells, **c** G-MDSC and **d** M-MDSC is shown in the tumor immune population (no significant differences were observed between control and treated tumors)

Flow cytometry data revealed increased tumor macrophage infiltration 24 h after trastuzumab treatment compared to controls (Fig. 2a). On Day 4, 24 h after the second dose of trastuzumab, the percent of macrophages making up the tumor-infiltrating immune cells increased from 30.52 ± 5.34% in control to 45.87 ± 2.1% in treated mice (*P* = 0.02). On Day 7, 96 h after two doses of trastuzumab, no significant difference was seen between control (29.97 ± 6.63%) and treated (20.31 ± 5.3%) tumors of that group (*P* = 0.43). On Day 7, 24 h following a third doses of trastuzumab, the macrophage population increased from 26.54 ± 6.54% in control tumors to 50.05 ± 5.14% in treated tumors (*P* = 0.03). Populations of dendritic cells (Fig. 2b), G-MDSC (Fig. 2c) and M-MDSC (Fig. 2d) were quantified in all treatment groups; however, no significant differences were observed.

### M1 macrophage phenotype increases in tumors over the course of trastuzumab treatment

**Fig. 3:**
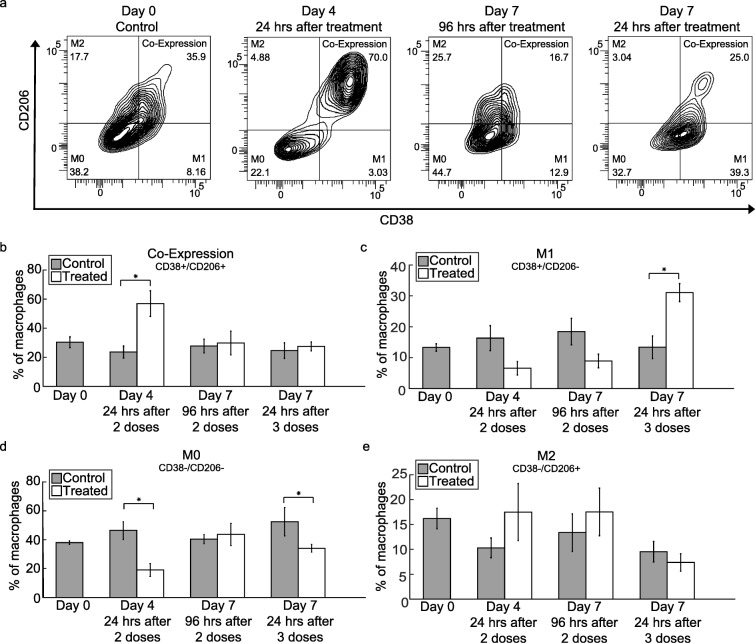
Macrophage phenotypes transition over the course of trastuzumab treatment. **a** Representative flow cytometry graphs of Day 0 control tumors and Day 4 and Day 7 trastuzumab treated tumors (96 and 24 h after treatment). Visual increases are observed in CD38+/CD206+ co-expressing macrophages on Day 4, 24 h after treatment and CD38+/CD206- M1 macrophages on Day 7, 24 h after trastuzumab treatment. **b** Percent of macrophages co-expressing CD38 and CD206 is shown revealing a significant increase (P = 0.02) from control in Day 4 treated tumors, after two doses of trastuzumab. **c** Percent of macrophages with an M1 phenotype (CD38+/CD206-) is shown revealing a significant increase (P = 0.02) in the M1 macrophage population in treated Day 7 tumors after three doses of trastuzumab. **d** Percent of macrophages with a non-differentiated, M0 phenotype (CD38−/CD206-) is shown revealing a significant decrease in population observed on Day 4 (P = 0.02) and Day 7 (P = 0.03), 24 h after treatment. **e** Percent of M2 macrophages (CD38−/CD206+) (no significant differences were observed between control and treated tumors)

Representative flow cytometry data plots of changes in M0 (CD38−/CD206-), M1 (CD38+/CD206-), M2 (CD38−/CD206+) macrophage subtypes and co-expression (CD38+/CD206+) over the course of trastuzumab treatment are shown in Fig. 3a [39, 40]. While control tumors were consistent between treatment groups, tumors co-expressing CD38 and CD206 increased on Day 4 (24 h after a second dose of trastuzumab) from 27.1 ± 4.12% to 56.9 ± 8.82% (*P* = 0.02) (Fig. 3b). No differences were seen in co-expression between control and treated tumors in the other treatment groups. After a third dose of trastuzumab was given, the percent of M1 macrophages significantly increased from 13.38 ± 3.65% to 31.07 ± 2.9% (P = 0.02) (Fig. 3c). There was a decrease in non-differentiated M0 macrophages on Day 4 from 45.36 ± 6.13% in control tumors to 19.03 ± 4.53% in treated tumors (P = 0.02) and on Day 7, 24 h after treatment from 52.44 ± 9.82% to 34.08 ± 2.74% (*P* = 0.03) (Fig. 3d). No significant differences were observed in the percent of M2 macrophages across treatment groups (Fig. 3e). In control and treated spleens taken down at Day 4, there were no statistical differences between macrophage populations (Supplementary Fig. 3) that suggests this effect is tumor specific.

### Vessel maturation index increases over the course of trastuzumab treatment and correlates with increasing M1 macrophage populations

**Fig. 4:**
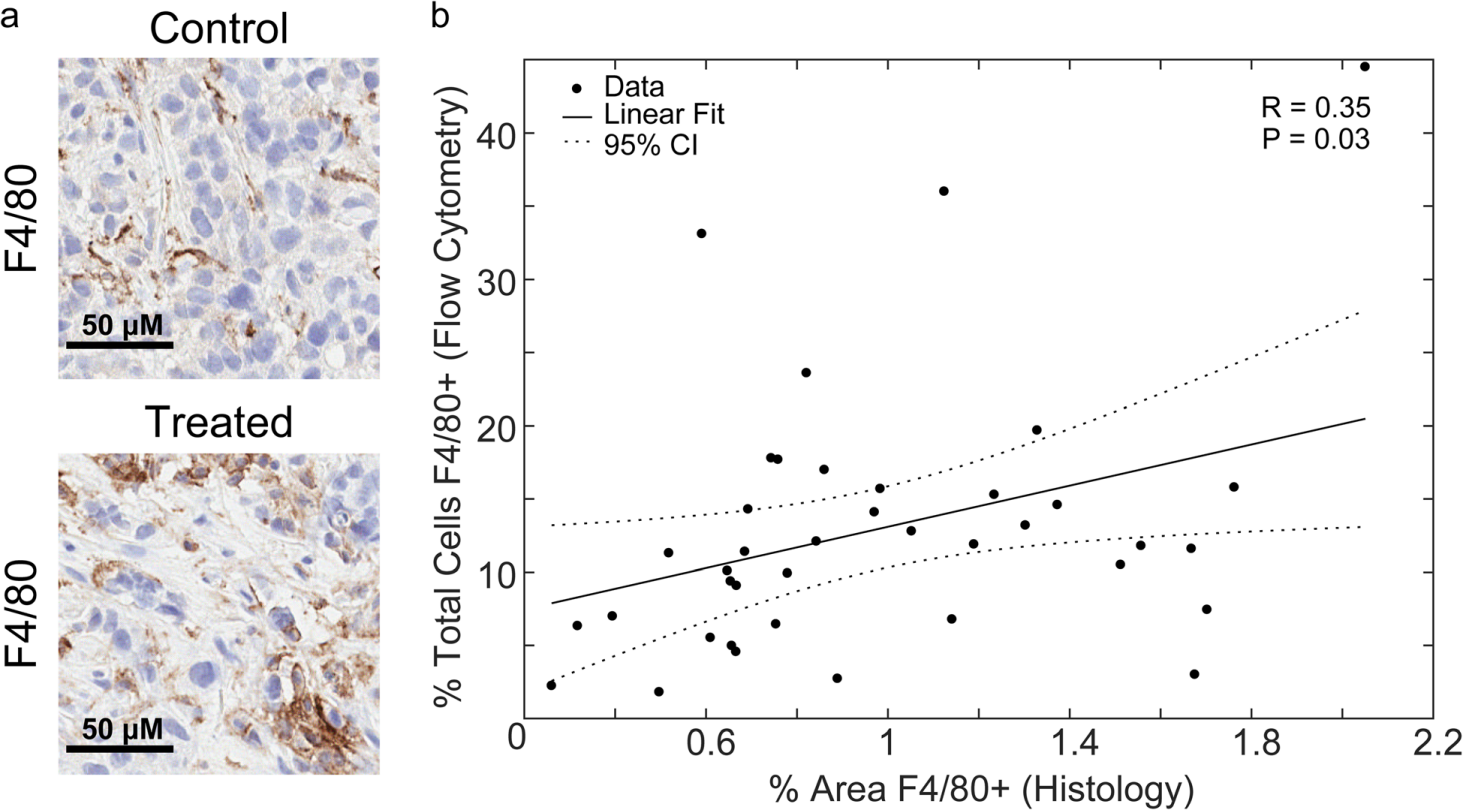
Correlation between F4/80+ determined by flow cytometry and IHC staining. **a** Representative IHC stained images of F4/80 in Day 4 control (top) and trastuzumab treated (bottom) tumors. **b** Linear correlation between percent of total cells F4/80+ as determined by flow cytometry and percent of total tumor area F4/80+ determined by IHC staining in the corresponding tumor central slice showing a positive linear correlation (R = 0.35, P = 0.03)

**Fig. 5:**
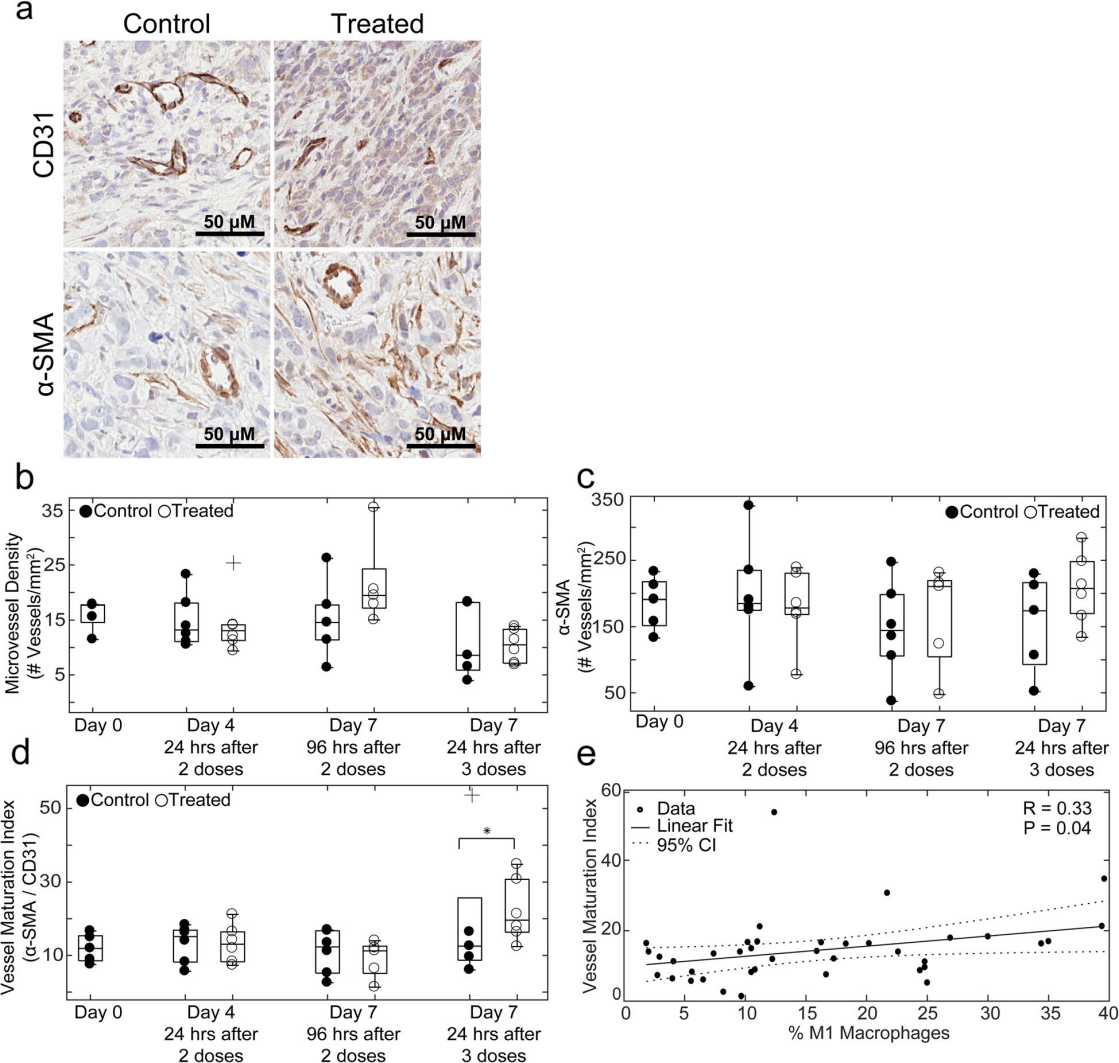
Vascular changes over the course of trastuzumab treatment positively correlate with macrophage phenotypic alterations. **a** Representative images of CD31 (top) and α-SMA (bottom) in Day 4 control and trastuzumab treated tumors. **b** CD31 microvessel density and **c** α-SMA coverage in control and trastuzumab treated tumors (no significant differences were observed). **d** Vessel maturation index as determine by ratio of α-SMA to CD31 microvessel density in control and trastuzumab treated tumors is shown, revealing a significant increase in vessel maturation index on Day 7, 24 h after a third dose of trastuzumab (*P* = 0.04). **e** Linear correlation between vessel maturation index and percent M1 macrophages in corresponding tumor halves as determined by flow cytometry showing a positive linear correlation (R = 033, P = 0.04)

Representative images of F4/80 staining in control and treated Day 4 tumors are shown in Fig. 4a. Comparison between percent F4/80+ cells in flow cytometry analysis and percent area F4/80+ in corresponding tumor central slices showed a significant positive linear correlation (R = 0.35, *P* = 0.03) (Fig. 4b). Representative images of CD31 and α-SMA in control and treated Day 4 tumors are shown in Fig. 5a. No significant differences in microvessel density or α-SMA coverage were observed between control and treated tumors (Fig. 5b and c, respectively). The vessel maturation index increased on Day 7 after three doses of trastuzumab from 11.13 ± 1.90% for control tumors to 22.58 ± 3.17% for treated tumors (*P* = 0.04) (Fig. 5d). Comparison of vessel maturation and percent M1 macrophages from flow analysis revealed a significant positive linear correlation (R = 0.33, P = 0.04) (Fig. 5e).

### Trastuzumab induced changes in inflammatory cytokines

**Fig. 6:**
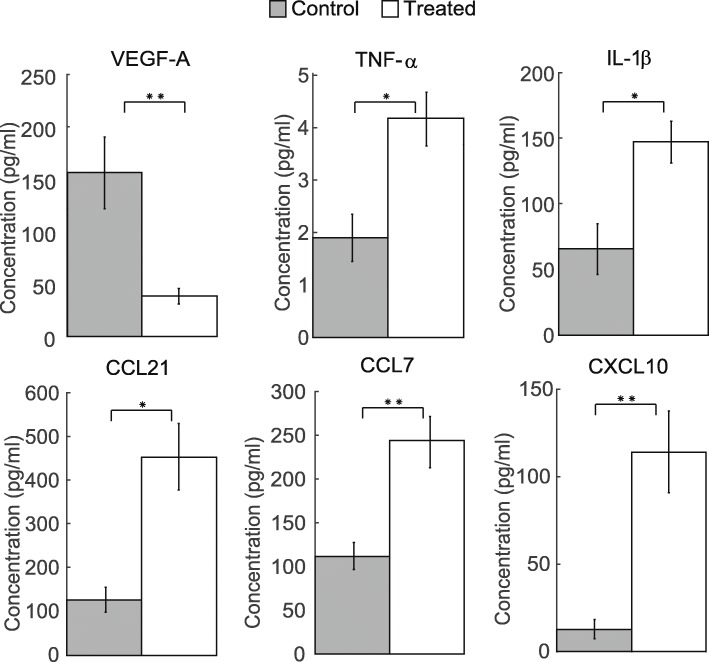
Continued trastuzumab treatment induces changes in tumor cytokine levels. Day 7 control and trastuzumab treated tumors (24 h after three doses of treatment) were evaluated for murine VEGF-A, TNF-α, IL-1β, CCL21, CCL7, and CXCL10 cytokine levels. A significant decrease in VEGF-A (*P* = 0.008) was observed in treated tumors compared to control. A significant increase in TNF-α (*P* = 0.024), IL-1β (*P* = 0.032), CCL21 (*P* = 0.016), CCL7 and CXCL10 (P = 0.008) was observed in treated tumors compared to control

To determine if cytokine differences were also present in tumors that displayed a higher percentage of M1 macrophages, a cytokine multiplex assay was conducted to evaluate differences between control and treated tumors on Day 7, 24 h after being treated with three doses of trastuzumab (Fig. 6). Treated tumors had a significant decrease in the pro-angiogenic factor VEGF-A (*P* = 0.008) and increases in pro-inflammatory cytokines TNF-α and IL-1β (*P* = 0.024 and 0.032, respectively). Significant increases in chemokines CCL21 (*P* = 0.016), CCL7, and CXCL10 (P = 0.008) were also seen in treated tumors compared to control. Changes in IL-6, IL-4, IL-10, IL-13, IL-23, and MMP-12 were evaluated, but no significant differences were found between control and treated tumors.

### Validation of treatment response and necrosis in control and treated tumors

**Fig. 7:**
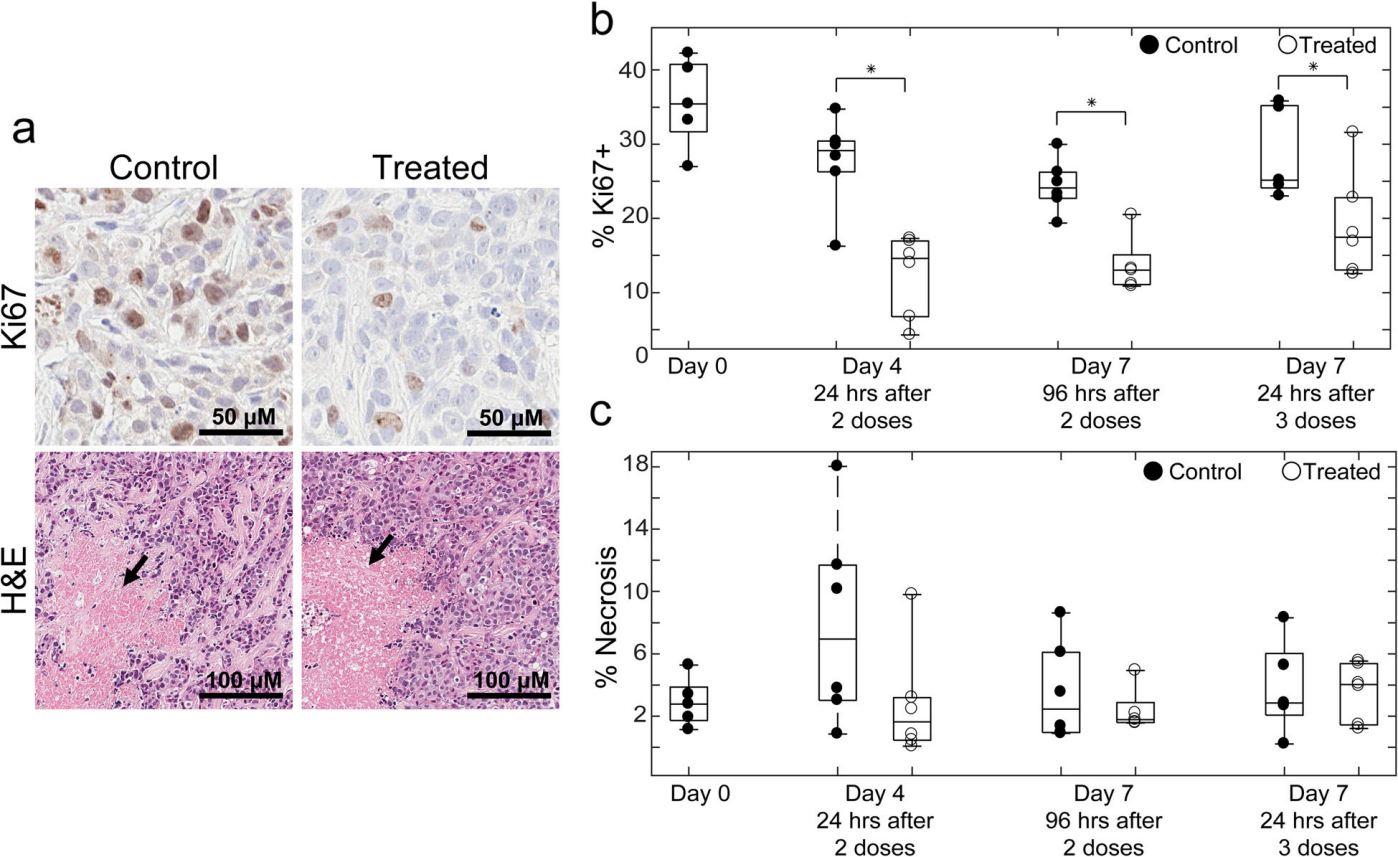
Quantitative analysis of Ki67 and necrosis in IHC stained samples. **a** Representative images of Ki67 (top) in control and treated Day 4 tumors are shown. Necrotic sections from H&E (bottom) indicated with black arrow in control and treated Day 4 tumors. **b** Percent nuclei Ki67+ in control and trastuzumab treated tumors. In all treatment groups, there was a significant decrease (*P* ≤ 0.05) in Ki67+ nuclei, validating treatment response. **c** Percent necrotic area in control and treated tumors (no significant differences were observed between control and treated tumors)

Representative images of Ki67 and H&E stains are shown in Fig. 7a. Treatment response was confirmed in all treatment groups indicated by significant decreases in Ki67 expression (Fig. 7b). Day 4 trastuzumab-treated tumors had an average Ki67 expression of 12.41 ± 2.06% while control tumors showed 27.66 ± 2.33% (*P* = 0.03). Treated tumors taken down on Day 7 after two doses of trastuzumab had 13.77 ± 1.56% Ki67+ nuclei compared to 24.40 ± 1.34% (*P* = 0.05) in control tumors. Treated tumors taken down on Day 7 after three doses of trastuzumab had 19.15 ± 2.66% Ki67+ nuclei compared to 28.70 ± 2.48% (P = 0.03) in Day 7 control tumors. No significant differences were observed between control and treated tumors in percent necrosis (Fig. 7c).

## Discussion

There is a well-developed field focused on exploiting vascular maturation to improve the response of cancers to therapy [11, 12, 22, 41]. Vascular maturation promoted by anti-HER2 targeted therapy with trastuzumab has been shown to enhance response to combination paclitaxel and doxorubicin chemotherapy in mouse models of breast cancer [7, 10]. Identifying underlying mechanisms of vascular changes will benefit clinical treatment regimens by elucidating methods to enhance and sustain vascular perfusion for improved drug delivery. Determining immune related mechanisms of vascular maturation could offer clinical benefit in developing combination immunotherapy treatments with trastuzumab. The present study evaluated trastuzumab-induced changes of myeloid cell infiltration in a murine model of HER2+ breast cancer. Our results demonstrated continuous trastuzumab treatment alters pro-inflammatory innate immune components of the tumor microenvironment while simultaneously increasing vascular maturation.

Immunophenotyping by flow cytometry revealed a transient increase in macrophage infiltration in treatment groups that were evaluated 24 h after administration of trastuzumab (Fig. 2a). With continued treatment, macrophages transitioned to an M1 phenotype (Fig. 3c). When treatment stopped for 4 days, macrophages reverted to phenotypes similar to those found in control tumors. The results from our study showed an increase in M1 macrophage population positively correlating with an increase in vascular maturation after persistent trastuzumab treatment (Fig. 5e). Similarly, in a murine model of pulmonary fibrosarcoma, Rolny et al. showed that genetically inducing tumor production of histidine-rich glycoprotein (HRG) inhibited tumor growth and metastasis, and HRG only sustained vascular normalization through the polarization of M1 macrophages [26]. Furthermore, Huang et al. found that low dose anti-VEGF receptor 2 therapy normalized tumor vasculature and polarized TAMs towards an M1 phenotype and improved response to combination immunotherapy in mouse models of breast cancer [25]. Additional studies depleting macrophages from the system used in our study would offer further quantitative information on macrophage contribution to vascular maturation and tumor response.

The changes in cytokine profiles of trastuzumab treated tumors support the results from immunophenotyping and histological evaluation. Our study revealed a decrease in host VEGF-A and simultaneous increases in several inflammatory cytokines and chemokines (Fig. 6), which agrees with what has been previously shown [7]. VEGF-A is a key regulator of angiogenesis and decreases in VEGF-A aligns with the increase in vascular maturation evaluated by histology with continued treatment of trastuzumab (Fig. 5d). In conjunction with an increase in total macrophage infiltration 24 h after treatment, there was a simultaneous decrease in non-differentiated, M0 macrophages (CD38−/CD206-) (Fig. 3d). This could in part be due to changes in inflammatory cytokines and chemokines in the tumor microenvironment (Fig. 6). M1 macrophages secrete increased amounts of TNF-α and IL-1β over M2 or M0 macrophages [33]. With continued treatment of trastuzumab, by Day 7 we see an increase in M1 macrophages (Fig. 3c) and increases in TNF-α and IL-1β (Fig. 6). Chemotaxis is induced differently in M1 and M2 macrophages. In this study, CXCL10, CCL21 and CCL7 were both upregulated in trastuzumab treated tumors. This is supported by other studies which have found CXCL10 and CCL21 to induce chemotaxis specifically in M1 macrophages [42, 43]. Studies show CCL7 can induce chemotaxis in both M1 and M2 macrophages [43, 44]. Although the changes in cytokines of the tumor microenvironment support the observed changes in macrophage phenotypes, many of the molecules can have both pro- and anti- tumor effects. Additional studies are needed to fully understand the underlying mechanisms cytokine treatments have to skew the inflammatory and angiogenic potential of tumors.

Clinically, TAMs are being investigated as prognostic and diagnostic biomarkers [45–47]. In several cancers, including breast, TAM density correlates with worse overall survival rates [47, 48]; however, TAM phenotypes can influence response to treatment. A recent study conducted in patients with stage II colon cancer showed that patients with a high M2/M1 (CD206+/CD68+) macrophage ratio were more likely to benefit from adjuvant chemotherapy than patients with a low M2/M1 ratio, who did not show any clinical benefit—therefore, identifying a subset of patients that would not need additional treatment following surgery [49]. Clinical trials with therapies inhibiting tumor macrophage recruitment to solid tumors have not been successful in showing improved tumor response [50, 51]. Ongoing preclinical and clinical work is being done in identifying drug targets that can reprogram immunosuppressive macrophages [52–54]. This study shows preliminary evidence that trastuzumab, already used in the clinic to treat HER2+ breast cancer, has the potential to repolarize M2 macrophages towards an M1 phenotype.

A limitation of this study is that mice were taken down at fixed time points and measurements could not be quantified longitudinally in the same animal. However, our methods allowed for an extensive immunophenotyping panel and simultaneous analyses of vascularity from central slice immunohistochemistry staining. Noninvasive imaging, such as immuno-PET imaging, is an alternative method that could give more finely time resolved data of immune infiltration after treatment, although it would only allow for measurement of one target at a time and may lack the spatial resolution required to fully assess immune response. Although a xenograft model was used in this study, murine macrophages do respond to trastuzumab (primarily through the FcγIV receptor [55]) and no significant differences in macrophage quantities or phenotypes were observed between spleens of Day 4 control and treated mice, showing immune response was directly tumor associated (Supplementary Fig. S3). The results from this study motivate future research in mouse models with intact (or humanized immune systems) to further characterize trastuzumab induced immune infiltration. The findings from this study would be strengthened with additional (similar) studies in other mouse models of HER2+ breast cancer (e.g., SKBR3, MDA-MB-361) as well as syngeneic models, such as MMTV-HER2/neu-transgenic mice. Determining effective markers to distinguish M1/M2 macrophage phenotypes is an active area of research [56, 57] and two other markers considered for this study were inducible nitric oxide synthase (iNOS, M1 expressed) and early growth response protein 2 (Egr2, M2 expressed) [56]. The distribution of macrophage phenotypes in Day 0 control spleens did not differ using iNOS and Egr2 compared to CD38 and CD206, and the latter were chosen for this study. Computational methods that analyze multidimensional flow cytometry data could be used in the future to analyze a panel with several macrophage markers to identify further phenotype subsets (i.e. M2a, M2b, M2c).

## Conclusions

In summary, this study identified novel differences in myeloid cell infiltration between control and trastuzumab-treated tumors in a murine model of HER2+ breast cancer as well as mechanistic reasoning of treatment induced vascular alterations. With continued treatment, M1 TAM phenotype increased and greater M1 populations correlated with increased vascular maturation. Previous studies show increases in therapeutic efficacy when optimizing trastuzumab combination dosing regimens with cytotoxic therapy. This study offers preliminary evidence of immune mechanisms of vascular maturation and the potential for trastuzumab to reprogram an immunosuppressive tumor microenvironment, specifically by polarizing macrophages towards an M1 phenotype. Further longitudinal assessments of trastuzumab induced immune changes may identify optimal combination regimens with immunotherapy and have potential to enhance clinical outcomes in breast cancer.

## Supplementary information

**Additional file 1 Supplementary Fig. 1.** Flow cytometry gating strategies to extract out populations of myeloid cells that were identified including macrophages, dendritic cells, granulocytic myeloid derived suppressor cells (G-MDSC), monocytic myeloid derived suppressor cells (M-MDSC) and M0, M1 and M2 macrophage phenotypes.

**Additional file 2 Supplementary Fig. 2.** Mutiplex protein concentrations were determined using corresponding standard curves for each analyte.

**Additional file 3 Supplementary Fig. 3.** Analysis of immune cell populations between control and treated mice showing no significant differences in macrophage quantities or phenotypes were observed between spleens of Day 4 control and treated mice.

## Data Availability

Data generated during this study are available from the corresponding author on reasonable request.

## Abbreviations

HER2: Human epidermal growth factor receptor 2
IHC: Immunohistochemistry
SMA: Smooth muscle actin
TAMS: Tumor associated macrophages
