# Supplementary figures and images for: Anti-HER2 induced myeloid cell alterations correspond with increasing vascular maturation in a murine model of HER2+ breast cancer
Source: BMC Cancer. 2020 Apr 28;20:359. doi: 10.1186/s12885-020-06868-4 (PMC7189470; doi:10.1186/s12885-020-06868-4)

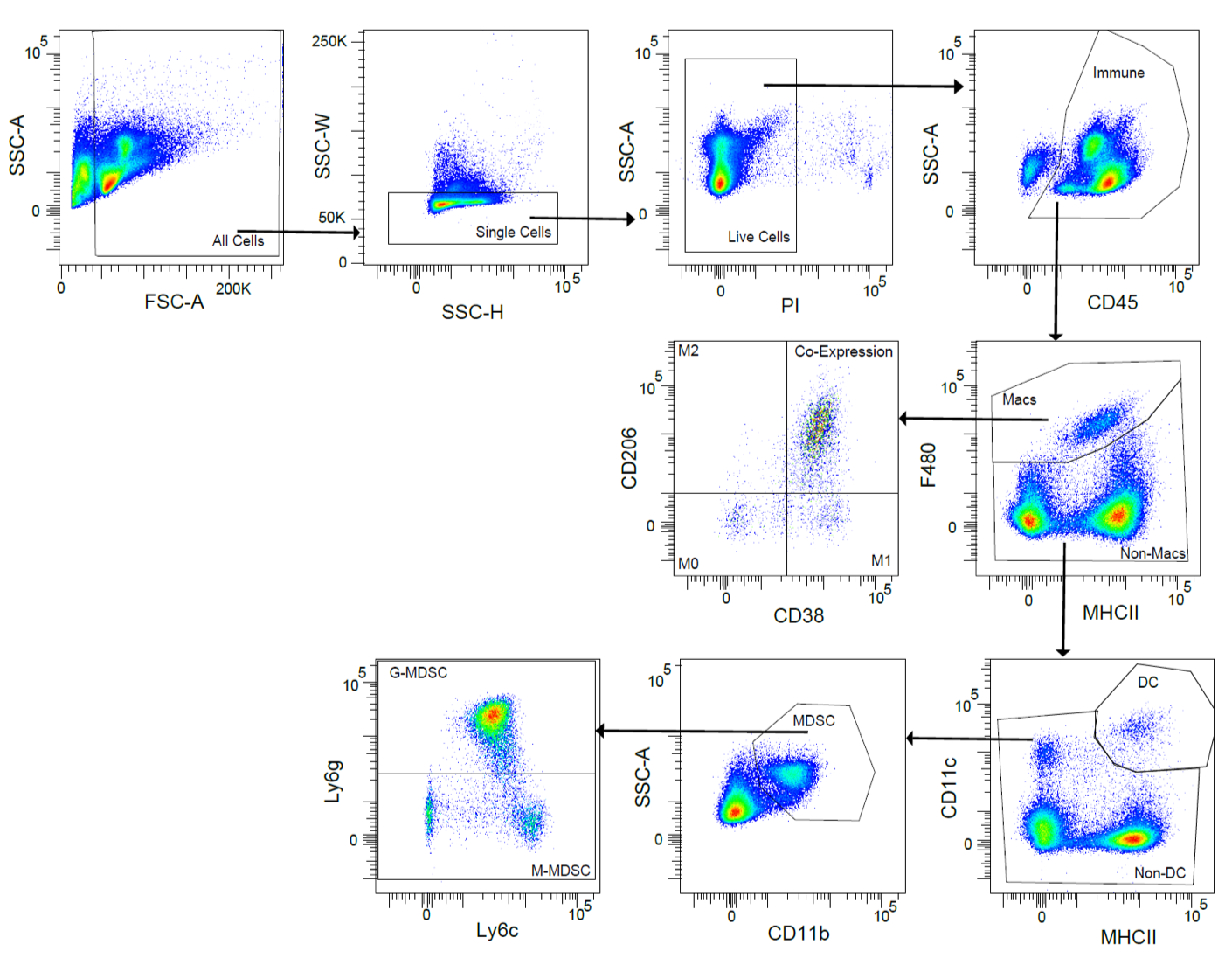

Supplement: Supplementary file 1 — Additional file 1 Supplementary Fig. 1. Flow cytometry gating strategies to extract out populations of myeloid cells that were identified including macrophages, dendritic cells, granulocytic myeloid derived suppressor cells (G-MDSC), monocytic myeloid derived suppressor cells (M-MDSC) and M0, M1 and M2 macrophage phenotypes. [file 12885_2020_6868_MOESM1_ESM.png]

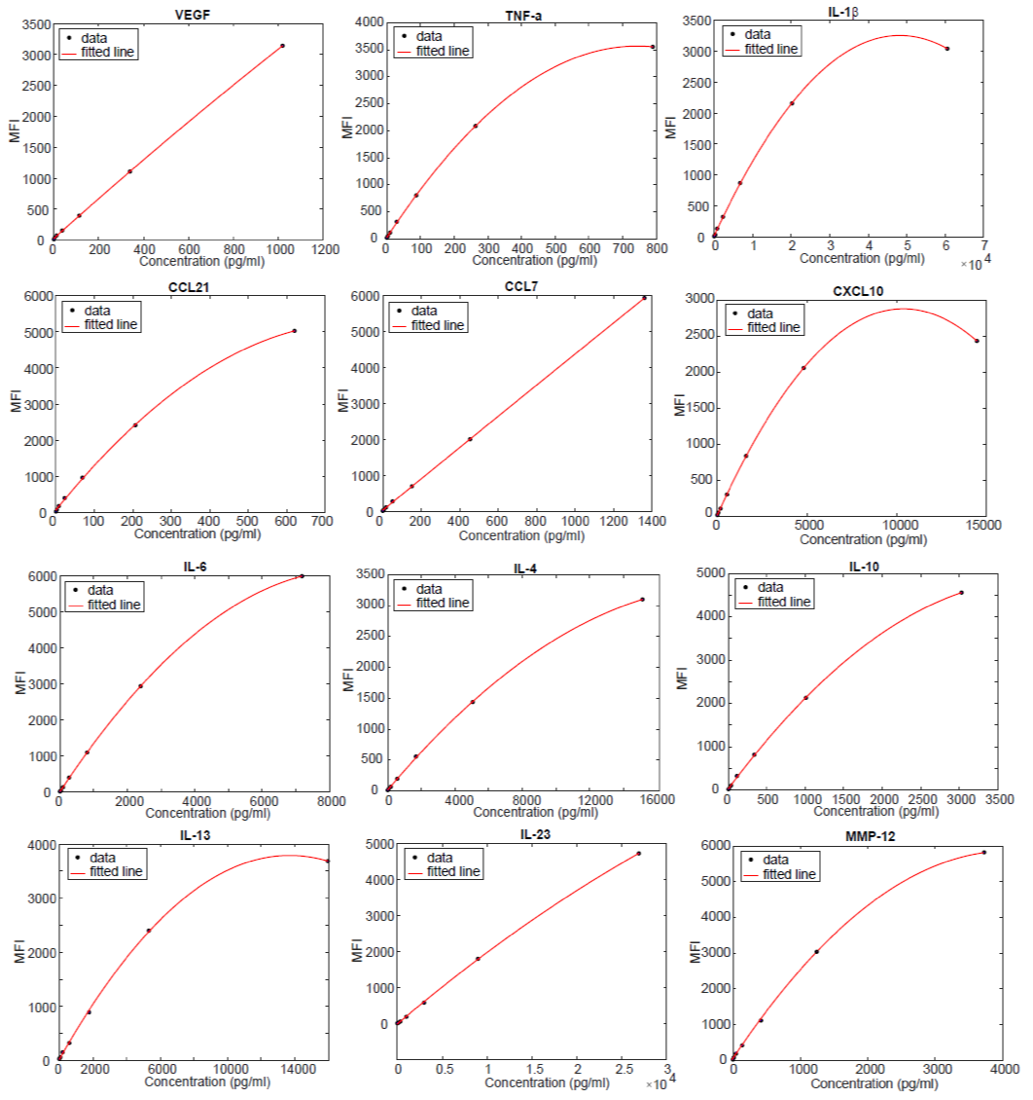

Supplement: Supplementary file 2 — Additional file 2 Supplementary Fig. 2. Mutiplex protein concentrations were determined using corresponding standard curves for each analyte. [file 12885_2020_6868_MOESM2_ESM.png]

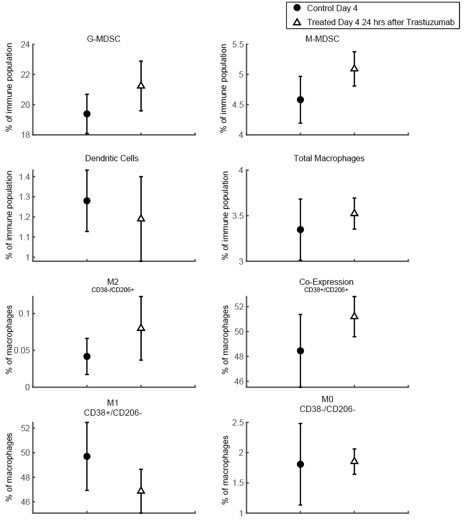

Supplement: Supplementary file 3 — Additional file 3 Supplementary Fig. 3. Analysis of immune cell populations between control and treated mice showing no significant differences in macrophage quantities or phenotypes were observed between spleens of Day 4 control and treated mice. [file 12885_2020_6868_MOESM3_ESM.jpg]
